# Estimation of forage biomass and vegetation cover in grasslands using UAV imagery

**DOI:** 10.1371/journal.pone.0245784

**Published:** 2021-01-25

**Authors:** Jérôme Théau, Étienne Lauzier-Hudon, Lydiane Aubé, Nicolas Devillers

**Affiliations:** 1 Department of Applied Geomatics, Université de Sherbrooke, Sherbrooke, Québec, Canada; 2 Sherbrooke Research and Development Centre, Agriculture and Agri-Food Canada, Sherbrooke, Québec, Canada; Northeastern University (Shenyang China), CHINA

## Abstract

Grasslands are among the most widespread ecosystems on Earth and among the most degraded. Their characterization and monitoring are generally based on field measurements, which are incomplete spatially and temporally. The recent advent of unmanned aerial vehicles (UAV) provides data at unprecedented spatial and temporal resolutions. This study aims to test and compare three approaches based on multispectral imagery acquired by UAV to estimate forage biomass or vegetation cover in grasslands. The study site is composed of 30 pasture plots (25 × 50 m), 5 bare soil plots (25 x 50), and 6 control plots (5 × 5 m) on a 14-ha field maintained at various biomass levels by grazing rotations and clipping over a complete growing season. A total of 14 flights were performed. A first approach based on structure from motion was used to generate a volumetric-based biomass estimation model (R^2^ of 0.93 and 0.94 for fresh biomass [FM] and dry biomass [DM], respectively). This approach is not very sensitive to low vegetation levels but is accurate for FM estimation greater than 0.5 kg/m^2^ (0.1 kg DM/m^2^). The Green Normalized Difference Vegetation Index (GNDVI) was selected to develop two additional approaches. One is based on a regression biomass prediction model (R^2^ of 0.80 and 0.66 for FM and DM, respectively) and leads to an accurate estimation at levels of FM lower than 3 kg/m^2^ (0.6 kg DM/m^2^). The other approach is based on a classification of vegetation cover from clustering of GNDVI values in four classes. This approach is more qualitative than the other ones but more robust and generalizable. These three approaches are relatively simple to use and applicable in an operational context. They are also complementary and can be adapted to specific applications in grassland characterization.

## 1. Introduction

Grasslands are among the most widespread ecosystems on earth. They occupy 26% of the land area and 70% of agricultural lands [1]. They represent a key element for global food security [2], in addition to providing ecological services related to erosion protection, wildlife habitat support, carbon sequestration and water harvesting [1]. Most of these ecosystems are in poor condition, mainly because of overgrazing [2]. Their management related to livestock grazing is a challenge because it involves complex spatio-temporal relationships between grazed plant species, animal behaviour and environmental factors [3, 4]. The tools and methods currently available for pasture monitoring rely mainly on field surveys that are time-consuming and difficult to generalize to the whole parcel [5, 6].

The development of precision farming over the last decades, and more specifically of the precision livestock farming concept, aims at increasing the use of advanced information and communication technologies in order to manage livestock more efficiently and dynamically [5, 7]. Remote sensing data are particularly interesting because they allow the acquisition of standardized and repeated information at different spatial and temporal scales. Satellite imagery or airborne imagery acquired by manned aircrafts have been tested in numerous studies to evaluate the botanical composition, structure, quality or quantity of grasslands [5]. Although they offer significant potential in agriculture, their relatively low spatial and temporal resolutions still limit their use for grasslands [5].

Recently, the increasing availability of unmanned aerial vehicles (UAV) and systems (UAS) offers opportunities for precise and frequent characterization of agricultural environments in a relatively accessible way [8]. The acquisition of visible and near-infrared imagery, which has been widely used in remote sensing for several decades to characterize vegetation, is becoming possible on small areas at an unparalleled level of spatio-temporal precision and without the limitations of satellite or manned airborne remote sensing [9]. Various biophysical parameters related to vegetation have already been estimated from UAV imagery, such as yield [10, 11], biomass [3, 12], LAI [13, 14], canopy height [4, 15], nutrient status [16, 17], and water stress [18, 19]. However, more studies focusing on the estimation of forage quantity and quality are needed.

Recent studies have attempted to predict biomass of forage parcels by testing the relationships between multispectral UAV imagery and biomass. The correlation levels obtained are highly variable and were between 0.01 and 0.93 [3, 4, 6, 20–24]. Although some of these studies obtained good predictive results, their application in an operational context has certain limitations related to the complexity of the processing chain and the integration of several environmental variables that may be unavailable (e.g. meteorological data, soil characteristics, topography). In addition, the potential for generalization of some studies is limited because they are based on approaches using site-specific data, which makes the relationships obtained difficult to transfer to other areas. Some studies also use data from a very limited range of time (e.g. fraction of the growing season), which limits the potential to predict biomass in these complex and dynamic environments [5]. Few studies have attempted to compare different approaches to characterize biomass. The majority used NDVI by default or only tested linear regression models. Relationships between vegetation indices (VIs) (e.g. NDVI) and biophysical parameters (e.g. biomass) are often non-linear and saturate at high values which affects the predictive potential of linear models [25, 26]. In addition, most studies have been conducted in arid and semi-arid environments, while grasslands in temperate climates are much less documented [5].

The purpose of this study is to test and compare several UAV-based multispectral image-processing approaches over an entire growing season in order to quantify forage biomass and vegetation cover in temperate-climate pastures.

## 2. Materials and methods

### 2.1. Study area

**Fig 1:**
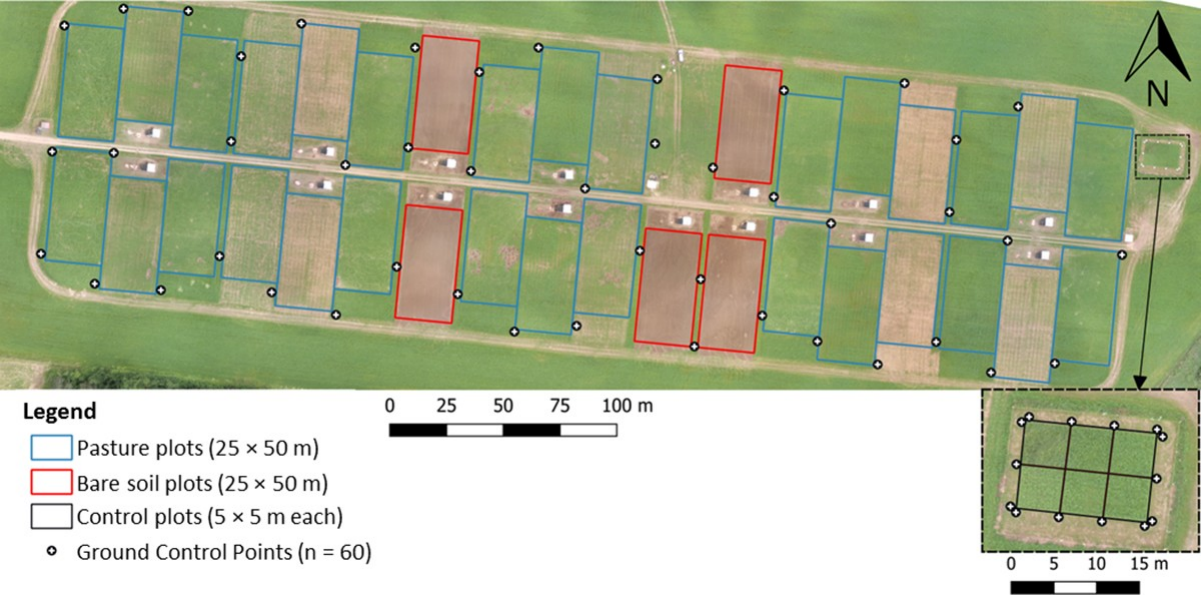
Map of the study area with 30 pasture plots, five bare soil plots and six control plots.

This study was conducted along with a larger study on nutrition and management of gestating sows in a pasture-based system [27]. The experiment was conducted in a 14-ha field located at the Sherbrooke Research and Development Centre of Agriculture and Agri-Food Canada (approximately 45°22' N, 71°50' W; QC, Canada). The study area includes silty-clay loam and sandy loam soils and is slightly inclined with an east-west slope varying between 191 m and 166 m above sea level. On this field, groups of three gestating sows were housed in two types of units (i.e. pasture or bare soil units) in which they were provided forage in addition to their feed (Fig 1). The year before the experiment, pasture plots were sown with a mix of red clover (*Trifolium pratense* L., 7 kg/ha), timothy (*Phleum pratense* L., 7 kg/ha), birdsfoot trefoil (*Lotus corniculatus* L., 5 kg/ha), meadow bromegrass (*Bromus commutatus* Schrad., 5 kg/ha), and Kentucky bluegrass (*Poa pratensis* L., 3 kg/ha). Each pasture unit consisted of three pasture plots (25 × 50 m), which the sows could access in rotation for periods of two weeks. Pasture plots were mechanically clipped to an approximate height of 10 cm after each grazing period and left undisturbed for regrowth for four weeks before sows came back. Ten groups of sows were housed in 10 pasture units, for a total of 30 pasture plots distributed across the field, and the grazing period was spread over the summer from mid-May to the end of September. In the same field, five additional groups of sows were housed in bare soil units each consisting of one bare soil plot (25 × 50 m) where forage was provided as hay *ad libitum* instead of pasture. These bare soil plots were superficially tilled with a harrow once a week to prevent weeds and grass from growing. Finally, in the eastern end of the field, a control unit of pasture composed of six control plots (5 × 5 m) was managed to collect data on biomass and forage growth over several durations during the summer (Fig 1).

### 2.2. Material

The UAS used in this study is a vertical take-off and landing unit equipped with a visible-near-infrared multispectral sensor and a visible sensor. The platform (Inspire 1 Pro, DJI, Shenzhen, China) has an endurance of 15 minutes and a maximum takeoff weight of 3,500 g. It is equipped with an on-board GNSS that automates the flights. The multispectral sensor used (Sequoia, Parrot, Paris, France) has four spectral bands: green (530–570 nm), red (640–680 nm), red edge (730–740 nm) and near infrared (770–810 nm), and has a resolution of 1.2 megapixels, which is equivalent to a ground sampling distance (GSD) of 11.3 cm at a height above ground level (HGL) of 120 m. The visible sensor used (Sequoia, Parrot, Paris, France) has a resolution of 16 megapixels, which is equivalent to a GSD of 3.3 cm at HGL of 120 m.

### 2.3. Data acquisition and processing

Three methods for the estimation of biomass or vegetation cover from UAV-based images were tested. One approach is based on regression between imagery-based volumetric measurements and field biomass data. Two approaches are based on vegetation indices calculated from multispectral imagery but using two different data processing methods (regression and classification).

#### 2.3.1. Image acquisition and processing

The study area was imaged during the summer of 2017 between June 6^th^ and September 26^th^, with one flight per week during the period of vegetation growth and one flight every two weeks at the end of the growing season (S1 Table). The dates of the flights were targeted in order to be synchronous with those of the sow rotation between pasture plots (i.e. Tuesday). A fixed flight time was also selected to limit variations in sun angle and illumination conditions. This target was not always met due to limiting weather conditions for UAV flights (e.g. rain, wind). Fourteen flights were made at an average height of 65 m above ground level, which represents an average GSD of 6.4 cm for the multispectral imagery and 1.7 cm for the visible imagery. The flight plans targeted 75% longitudinal and lateral overlaps. Ground control points (GCPs) were obtained using 60 targets distributed throughout the study area (Fig 1) and localized using a high-precision GNSS receiver in real time kinematic mode (RTK) (R8, Trimble, Sunnyvale, CA, USA) with an average accuracy of 1 to 2 cm in position and 2 to 4 cm in altitude.

For each acquisition date, multispectral and visible imagery were processed using the same procedure to produce an orthomosaic and a digital surface model (DSM). The Pix4D software (Pix4D SA, Lausanne, Switzerland) was used to perform the different steps of aerial triangulation using the 60 high-precision GCPs, bundle block adjustment, sparse matching, and dense matching to produce orthomosaics and DSMs [28]. Radiometric corrections were applied to convert images into radiance [29].

#### 2.3.2. Volumetric-based biomass model

This method used an estimation model based on a linear regression between volumetric calculations and biomass values. This method was only tested on the control unit due to the unavailability of a high-precision digital terrain model (DTM) on the rest of the study area. This area was managed to obtain biomass samples ranging from 0 to 6 weeks of growth within a control set up in order to obtain an evenly distributed variation of the biomass data. The control unit was divided into six control plots (5 × 5 m each) where biomass was measured after several durations of forage growth after clipping (1 duration/control plot: 0, 1, 2, 3, 4 and 6 weeks) repeated twice during the summer (May/June and July/August). On July 4^th^ and August 29^th^, three quadrats per control plot were sampled to estimate the average biomass of each control plot. For each quadrat, an area of 0.25 m^2^ of forage was cut at approximately 7 cm from the ground using electric grass shears. Samples were weighed and then dried at 55°C for at least 72 h and weighed again to determine fresh and dry-matter biomass. Biomass was then calculated on a fresh or dry-matter basis in kg/m^2^. The average biomass value for each control plot (n = 12 biomass values; 6 plots × 2 dates) was used for the volumetric estimation model.

A high-precision DTM was generated using the inverse distance weighted (d = 3) interpolation using the 14 high-precision GNSS points distributed at the edge of the control unit (Fig 1). Volumetric data were computed by subtracting DSM values from the DTM to obtain height differences for every pixel. The height value of every pixel was then multiplied by the pixel area to obtain a volume raster. The linear regressions for fresh and dry biomass were calculated between volumetric data and the 12 biomass values described above as well as root mean square error (RMSE) and normalised RMSE (NRMSE = RMSE/mean×100).

#### 2.3.3. Vegetation index selection

The relationship between several vegetation indices (Table 1) calculated on multispectral orthomosaics and biomass was done on the same set of 12 biomass values collected from the control unit and used for the volumetric-based biomass model. This selection of VIs is based on a literature review applied to biomass estimation as well as on the availability of spectral bands of our sensor. Correspondence between the average biomass from each control plot and the average index value of a 3.5 × 3.5 m polygon extracted from the same control plots was studied for fresh and dry biomass separately. Non-linear regression was calculated between the tested indices and fresh and dry biomass. Regression equation was reversed and predicted biomass values were calculated. Linear regression between predicted values and measured biomass values was calculated, and coefficient of determination was determined as a measure of the quality of the prediction. The best vegetation index was selected for the next step based on two criteria:

1. Level of variation in the data based on the coefficient of variation (CV). A vegetation index with a greater variability for a same set of biomass samples would have a higher discriminating power;
2. Quality of the regression for both fresh and dry biomass based on the value of the coefficient of determination of the linear regression between predicted and observed biomass values;

**Table 1:** Vegetation indices tested for biomass estimation using 12 biomass values from the control unit, range of values observed (minimum and maximum values) and selection criteria: Coefficient of variation (CV) and regression quality (coefficient of determination (R^2^) of the linear regression between predicted and observed values for fresh (F) and dry (D) biomass).

Index	Formula [reference]		Min	CV (%)	R^2^	R^2^
			Max		F	D
NDVI^a^	$\frac{\left(NIR-Red\right)}{\left(NIR+Red\right)}$	[30]	-0.02	55.4	0.50	0.45
			0.85			
GNDVI^b^	$\frac{\left(NIR-Green\right)}{\left(NIR+Green\right)}$	[31]	-0.01	61.6	0.77	0.75
			0.56			
MSAVI2^c^	$\frac{2\cdot NIR+1-\sqrt{{\left(2\cdot NIR+1\right)}^{2}-8\cdot (NIR-Red)}}{2}$	[32]	13.1	30.1	0.60	0.61
			31.4			
OSAVI^d^	$\frac{\left(1+0.16)\cdot (NIR-Red\right)}{\left(NIR+Red+0.16\right)}$	[33]	-0.02	55.2	0.48	0.43
			0.98			
SAVI^e^	$\frac{\left(1+0.5)\cdot (NIR-Red\right)}{\left(NIR+Red+0.5\right)}$	[34]	-0.03	55.5	0.49	0.44
			1.26			
TVI^f^	0.5⋅(120⋅(NIR−Green))−200⋅(Red−Green)	[35]	-72	57.1	0.32	0.32
			2629			
NDRE^g^	$\frac{NIR-RE}{NIR+RE}$	[36]	21.6	28.1	0.42	0.44
			52.0			
LogR^h^	log$\left(\frac{NIR}{RED}\right)$	^i^	-0.04	61.5	0.37	0.36
			2.53			
Datt1	$\frac{\left(NIR-RE\right)}{\left(NIR-Red\right)}$	[37]	0.02	56.1	0.70	0.76
			0.29			

^a^ Normalized Difference Vegetation Index

^b^ Green Normalized Difference Vegetation Index

^c^ Modified Soil Adjusted Vegetation Index-2

^d^ Optimized Soil Adjusted Vegetation Index

^e^ Soil Adjusted Vegetation Index

^f^ Triangular Vegetation Index

^g^ Normalized Difference Red-edge

^h^ Log Ratio

^i^ Index Database: https://www.indexdatabase.de/

Results of this analysis are presented in Table 1. The Green Normalized Difference Vegetation Index (GNDVI) showed the highest variation for the biomass studied (CV = 61.6%) and the highest coefficients of determination for both fresh and dry biomass (0.77 and 0.75, respectively). This index was therefore selected for the next steps.

#### 2.3.4. GNDVI-based biomass model

**Fig 2:**
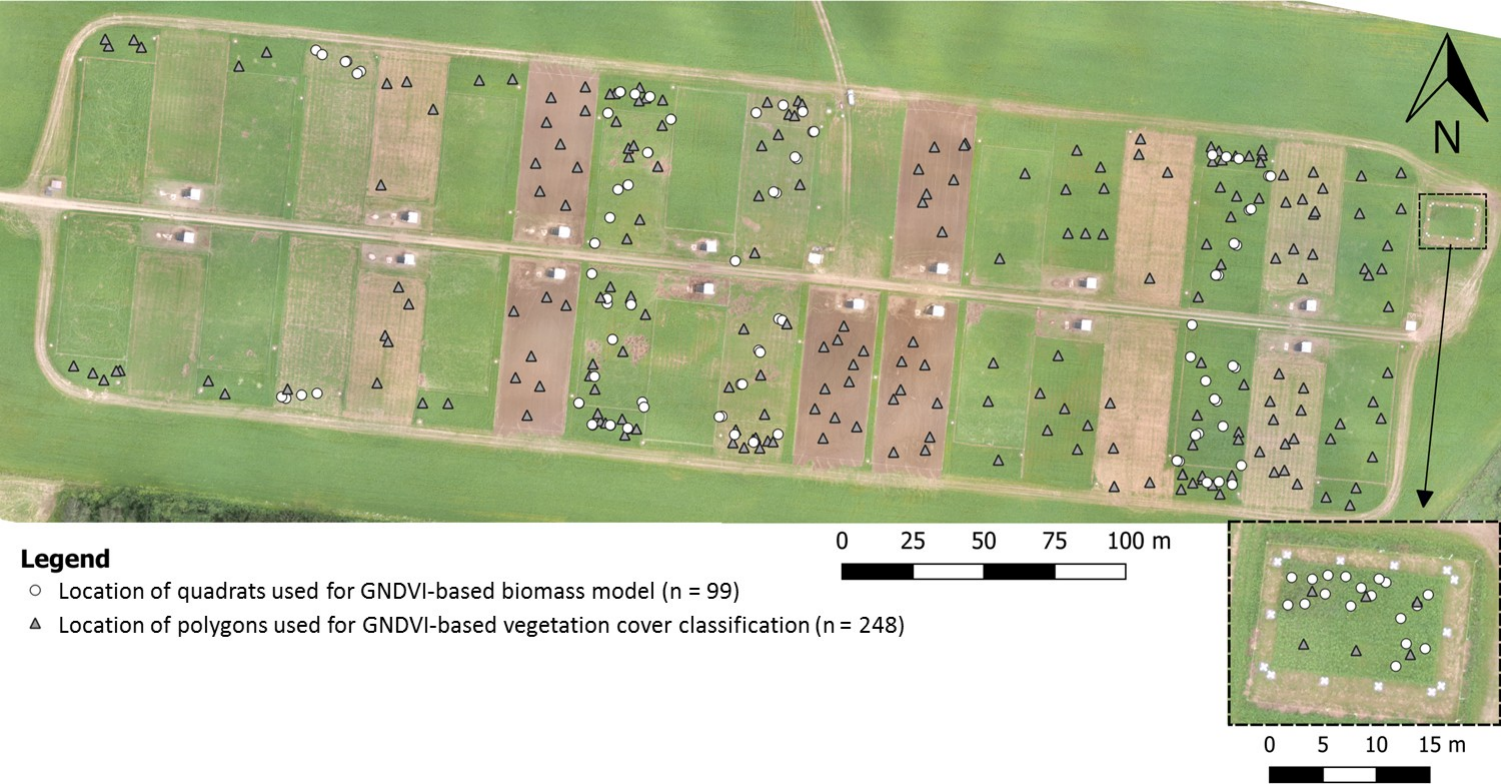
Location of samples used for GNDVI-based biomass model and vegetation cover classification.

This method tested an estimation model using non-linear regression between vegetation index and biomass values to predict biomass. A set of 99 biomass quadrats was collected at various times and locations in the field from the beginning of July to mid-September to determine and validate the biomass estimation model (Fig 2). In order to maximize the variability of the data set, quadrats selection was stratified. Specific plots corresponding to growing stage between 2 and 6 weeks were selected in the pasture and control units. Three to five quadrats were sampled in each selected plot for each growing stage. For each quadrat, biomass was measured using the method described in section 2.3.2.

A subset of 49 data points (Table 2) was randomly selected from this dataset and used as a training dataset to calculate prediction equations for fresh and dry biomass from non-linear regressions on GNDVI values using PROC NLIN in SAS® software (Statistical Analysis System, Release 9.4, 2002–2012. SAS Institute Inc., Cary, NC). Concordance analyses (i.e. linear regression) between the predicted and actual biomass values of this training dataset were carried out to determine the precision of the model. Central tendency error, regression error and residual error were calculated as well as RMSE and NRMSE. The remaining 50 data points from the dataset were used as a validation dataset (Table 2). A second concordance analysis was conducted to compare observed and predicted values of the validation dataset in order to test the potential of the model for generalization. Central tendency, regression and residual errors, as well as RMSE and NRMSE, for the prediction of fresh and dry biomasses were also calculated.

**Table 2:** Number and characteristics of biomass quadrats (0.5 × 0.5 m) collected in the field during the growing season and used for the GNDVI-based biomass estimation model.

Data set	Characteristics	Fresh biomass	Dry biomass
		(g/m^2^)	(g /m^2^)
Training	Mean ± SD	1924 ± 970	354 ± 172
n = 49	Min / Median / Max	208 / 1724 / 4708	60 / 324 / 824
Validation	Mean ± SD	1891 ± 944	348 ± 175
n = 50	Min / Median / Max	336 / 1784 / 4600	76 / 316 / 820

#### 2.3.5. GNDVI-based vegetation cover classification

This method used vegetation index classification to determine vegetation cover. A set of 248 3.5 × 3.5m polygons (Fig 2) was selected pseudo-randomly from the aerial imagery during the scheduled flights. Polygon selection was stratified by forage growth using the pasture rotation schedule representing various levels of vegetation cover. Polygons were selected in bare soil, pasture and control plots throughout the field for forage growth durations ranging from 1 to 6 weeks across the season (a detailed description of polygon selection is provided in S2 Table). Because GNDVI data were not normally distributed, further work of classification was done on the transformed variable e^GNDVI^. Values of e^GNDVI^ were extracted for each pixel within each polygon and averaged using the open source geographic information system QGIS (version 3.4, www.qgis.org). A cluster analysis was performed to categorize mean e^GNDVI^ values with a maximum of four classes using the FASTCLUS procedure in SAS®. A discriminant analysis was then performed using the DISCRIM procedure in SAS® to determine the boundaries between these classes. The four classes of vegetation cover obtained were then described *a posteriori* (see section 3.3), according to the season (date) and the stage (duration in weeks) of forage growth of the polygons falling within each class.

## 3. Results

### 3.1. Volumetric-based biomass model

**Fig 3:**
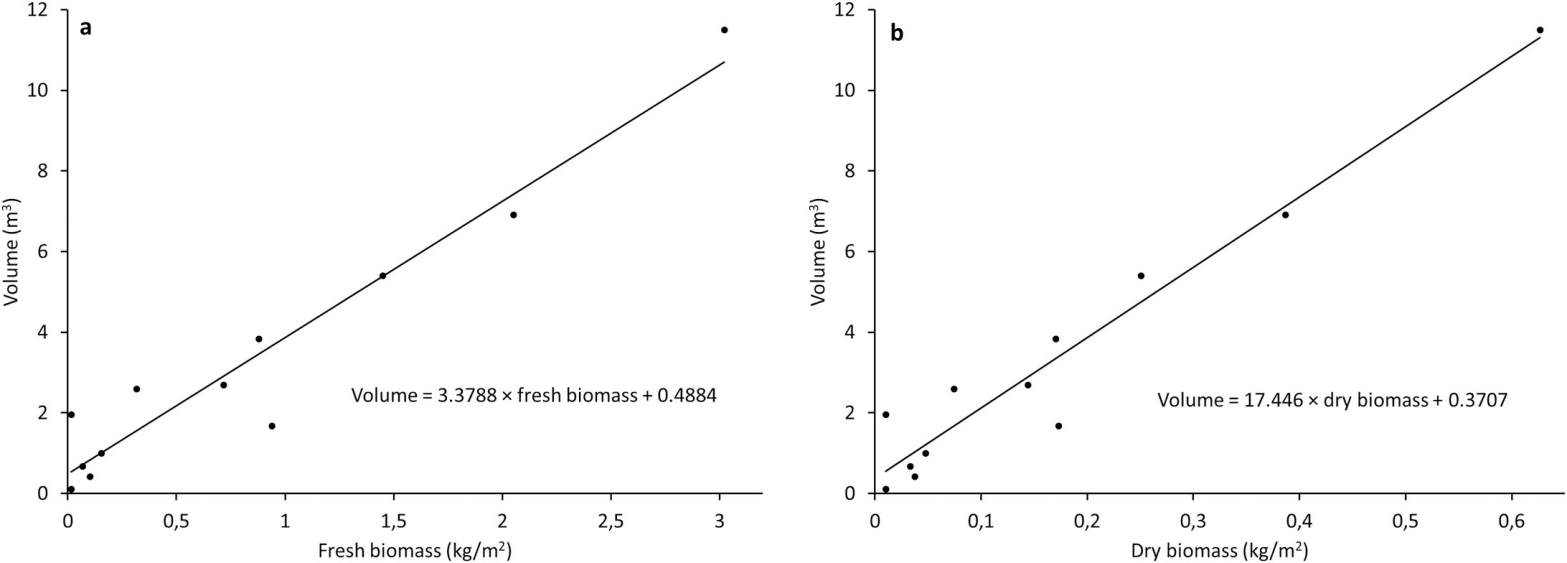
Linear regressions between volumetric values and fresh (a) and dry (b) biomass (n = 12).

The linear regressions between volumetric values and fresh and dry forage biomass are presented in Fig 3A and 3B. Results produced comparable and high coefficients of determination (R^2^) values of 0.93 and 0.94 for fresh and dry biomass, respectively. Root mean square errors and NRMSEs were 0.072 kg/m^2^ and 8.9% for fresh biomass and 0.013 kg/m^2^ and 7.9% for dry biomass, respectively.

### 3.2. GNDVI-based biomass model

**Fig 4:**
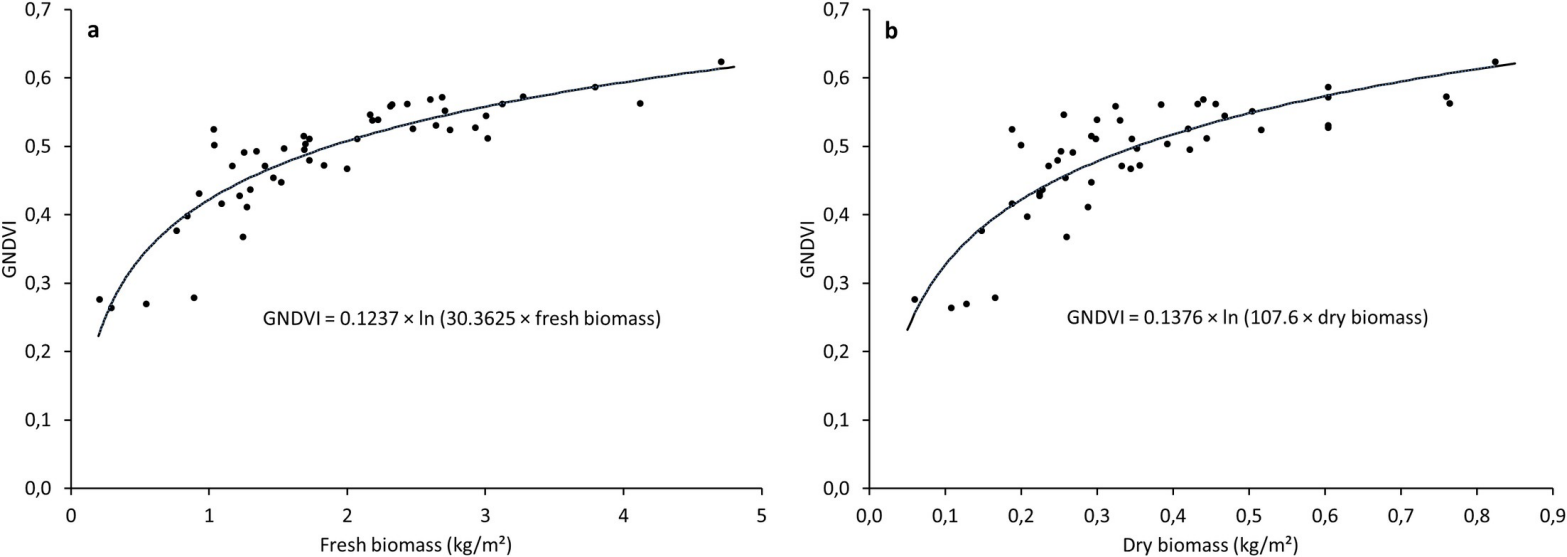
Non-linear regressions between GNDVI values and fresh (a) and dry (b) biomass (n = 49).

The non-linear regressions between GNDVI values and fresh and dry forage biomass on the training dataset are presented in Fig 4A and 4B.

Regression equations were reversed to determine prediction equations as follows:

$$Fresh\;biomass=e^{\left(GNDVI/0.1237\right)}/30.3625$$ (1)

$$Dry\;biomass=e^{\left(GNDVI/0.1376\right)}/107.6$$ (2)

where Fresh biomass and Dry biomass are expressed in kg/m^2^.

The concordance analyses between predicted and observed values of the training dataset (not represented) gave RMSEs of 0.470 and 0.102 kg/m^2^ and NRMSEs of 24 and 29% for fresh and dry biomass, respectively. The coefficients of determination of the linear regressions (R^2^) were 0.80 and 0.66 for fresh and dry biomass, respectively. Central tendency error and regression error were 1.56% and 1.25% for fresh forage and 0.998% and 8.86% for dry forage, respectively.

**Fig 5:**
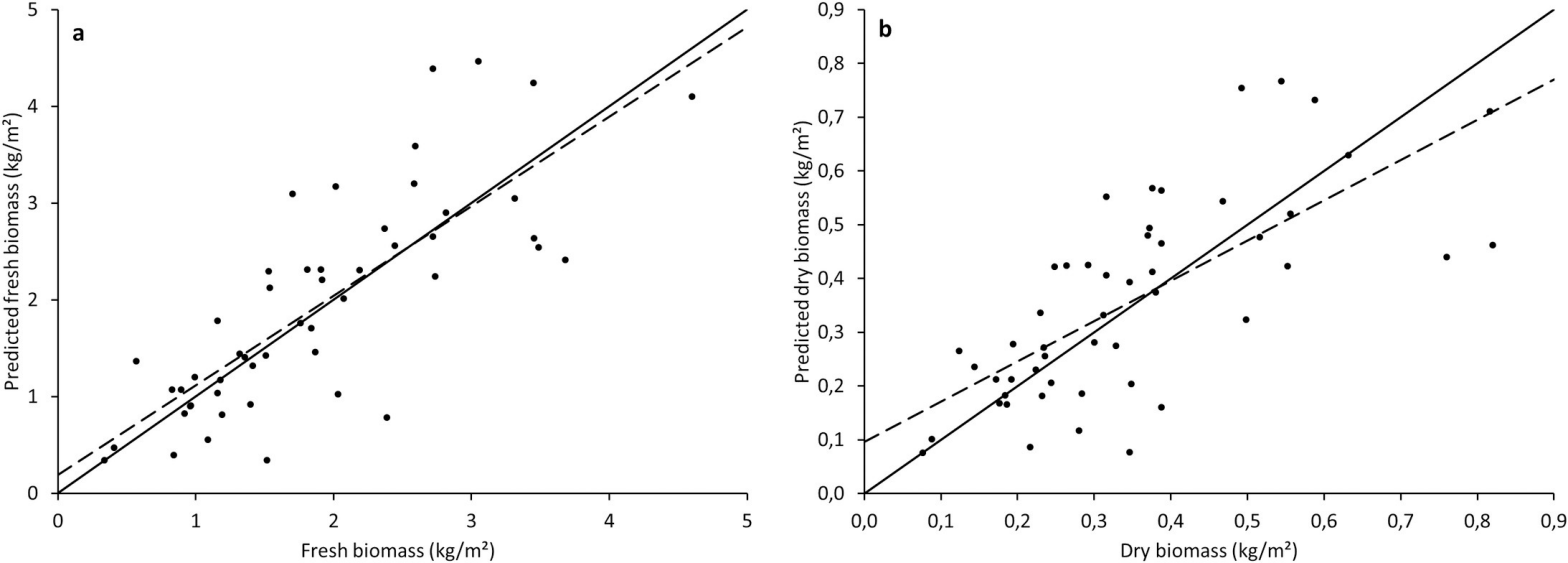
Concordance analysis between observed and predicted values for fresh (a) and dry (b) biomass of the validation dataset (n = 50). Continuous line = 1:1 line, dashed line = linear regression.

In order to validate the model, concordance analyses between predicted and observed values were done on the validation dataset and are presented in Fig 5A and 5B.

Central tendency error and regression error, which explain the quality of the model (the lower the better), were 0.56% and 1.04% for fresh forage and 0.43% and 10.04% for dry forage. The coefficients of determination of the linear regressions were 0.63 and 0.50 for fresh and dry forage, respectively. The RMSEs, which measure the precision of the model, were 0.682 kg/m^2^ for fresh forage and 0.132 kg/m^2^ for dry forage, corresponding to NRMSEs of 36 and 38% respectively.

### 3.3. GNDVI-based vegetation cover classification

**Fig 6:**
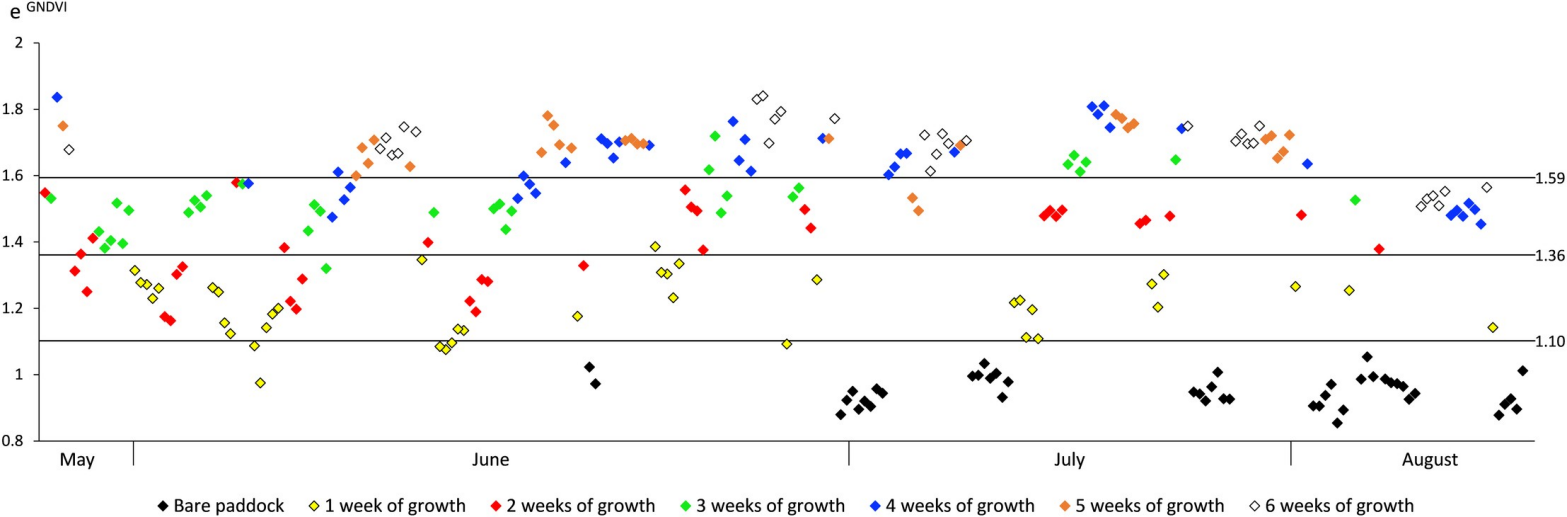
Values for average e^GNDVI^ from polygons selected in bare soil and pasture units during the entire growing season and for different duration of forage growth (n = 248).

The three thresholds between the four classes of vegetation cover obtained from the cluster analysis on the average e^GNDVI^ values for the 248 polygons are 1.10, 1.36 and 1.59. Fig 6 presents the average e^GNDVI^ values of the 248 polygons in relation to the type of unit (i.e. pasture unit or bare soil unit), the season (e.g. date of clipping, i.e. start of growth) and stage of forage growth (i.e. duration in weeks). Fig 6 shows each threshold between classes and how polygons are distributed across the season for growth durations from one to six weeks. As growth duration increases, polygons present higher e^GNDVI^ values, and as season progresses, forage growth slows down and e^GNDVI^ values decrease. Based on this information, the four classes of vegetation cover can be described as follows:

1. Bare soil: e^GNDVI^ < 1.10, corresponding mainly to sites in bare soil units;
2. Low vegetation: 1.10 ≤ e^GNDVI^ < 1.36, corresponding to one week of forage growth across all season, and two weeks of forage growth in early season (May, June);
3. Medium vegetation: 1.36 ≤ e^GNDVI^ < 1.59, corresponding to three weeks of growth in early season, to two weeks of growth in July and to two to six weeks of growth in late season (August, September);
4. High vegetation: e^GNDVI^≥ 1.59, corresponding to more than three weeks of growth in early season and more than two weeks of growth in July.

### 3.4. Visual comparison of the three estimation methods

**Fig 7:**
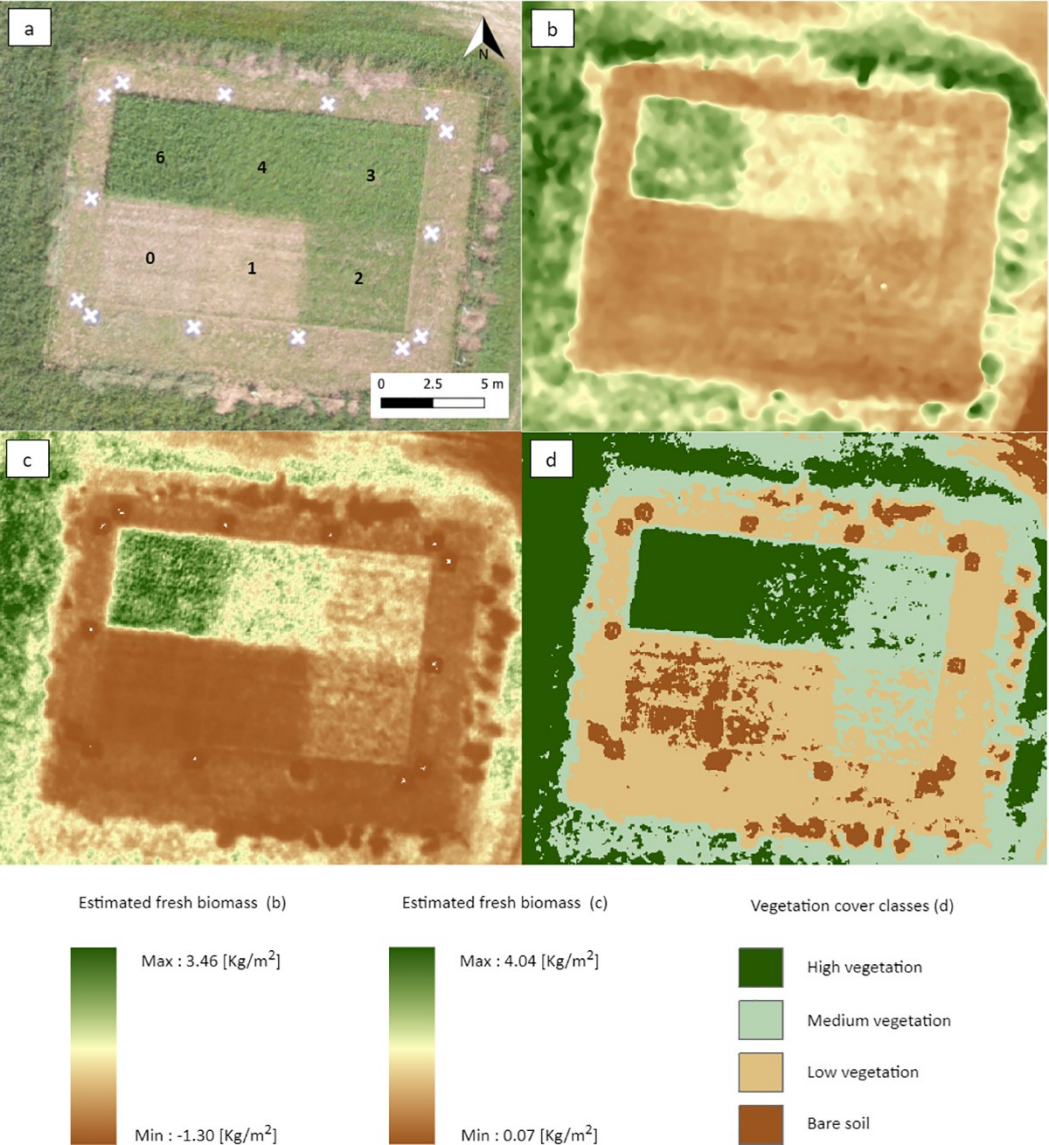
Visual comparison of three biomass or vegetation cover estimation methods for the imagery acquired on August 29^th^ in the control unit: (a) the RGB imagery including the forage growth duration per plot (weeks), (b) the volumetric-based biomass model, (c) the GNDVI-based biomass model, and (d) the GNDVI-based vegetation cover classification.

A comparison of the three methods is presented in Fig 7 for the control unit. These results show that similar patterns can be observed between the three approaches, which illustrates the convergence of the methods. However, the six forage growth durations (0, 1, 2, 3, 4 and 6 weeks) are not clearly observable for all methods. While weeks 2, 3, 4 and 6 are evident in the three methods, weeks 0 and 1 are more difficult to distinguish for GNDVI-based and volumetric-based biomass approaches.

Fig 7 also illustrates the difference in the level of generalization between the methods. The classification-based approach provides a higher level of generalization than the other two methods, which provide more details between pixels. A comparison with the RGB image also makes it possible to qualitatively assess the performance of the three methods. The results seem to adequately and comparably describe the vegetation patterns inside the control unit.

Fig 7 also provides relevant information regarding elements not planned in the experimentation. Small patches of dead vegetation (piles of dead vegetation from the maintenance of this area), observable along the outside limits (north, east, and south) of the control unit, seem to be well characterized by the GNDVI-based approaches (patches of low values), while the volumetric-based approach shows a wide range of values in these areas. It illustrates a limit of the volumetric approach, which is based on the positive relationship between volumetric values and field biomass. Volumes calculated on these piles of dead vegetation were then converted into living biomass values.

## 4. Discussion

### 4.1. Contributions of the study

This study compares three processing approaches based on visible and near-infrared imagery acquired by a UAV for the estimation of grassland biomass or cover. This study provides five original contributions compared to the existing literature.

1. To our knowledge, few studies have carried out comparisons of approaches in the same territory to characterize the biomass in grasslands, pastures, or forage fields. Viljanen et al. [23] or Michez et al. [4] compared approaches based on vegetation height, reflectance and vegetation indices, and a combination of the two. Cooper et al. [38] compared volumetric estimations based on imagery photogrammetry with terrestrial laser scanning and disc pasture meter measurements to estimate biomass. By eliminating the variability linked to the study site (e.g. growth conditions, phenological stage, cultivated varieties), the comparative approach makes it possible to assess the performance of each approach on the same basis and to highlight their potential in various applications and their limitations.
2. The majority of similar approaches for characterizing biomass in grasslands, pastures, or forage fields are based on individual values of spectral bands (i.e. digital number or reflectance values) [21], vegetation indices [3, 6, 20, 22, 39], canopy/crop height models [4, 38, 40–42], and multi-source models [4, 23, 24, 43]. To our knowledge, no study has tested the use of classification to categorize the level of vegetation cover in this type of environment. As for the volumetric-based approach, it has been used recently [4, 23, 38, 40, 41, 43, 44] and provides variable but promising results. In this study, these two approaches demonstrate significant and complementary potential for biomass characterization.
3. The majority of similar studies are based on data covering a fraction of the growing season, with periods ranging from one day [22] to a few days [4, 21, 23], or a few days distributed over the growing season [6, 20, 39, 42]. Few studies acquired data frequently over the entire growing season [3, 24, 44] as is the case with this study. However, some studies applied different levels of fertilization on their study plots, which provided different stages of growth for the same date [22, 23, 42]. The frequent acquisition of data distributed throughout the growing season ensures a wide variability of the dataset and facilitates the generalization of the models to other sites.
4. Very few studies have compared the efficiency of multiple indices. Regression analyses between biomass and vegetation index (VI) values showed medium to high coefficient of determination ranging from 0.6 to 0.9 for VI such as NDVI, RVI, RDVI, OSAVI, MSAVI, SAVI [20], RGBVI, NGRDI, GLI, VARI, SR [22], GRVI, GNDVI and NDRE [4], or GRVI, MGRVI, RGBVI, OSAVI, ExG, ExR [23]. In the present study, nine VIs were compared for biomass estimation. Among studies using VIs, NDVI and RVI were the most frequently used and most often chosen by default. Results of this study showed that GNDVI and Datt1 were the indices with the highest coefficient of determination (≥0.7), whereas NDVI, which is the most commonly used index, had a coefficient near 0.5 only. Unlike most studies using UAVs for grasslands characterization, we also tested nonlinear models that appear to be more representative of the non-linear relationship between VIs and biophysical parameters [25, 26]. This illustrates the importance of testing different VIs in order to identify those that present optimal results for the type of environment considered. The sensitivity and saturation of VIs linked to the soil reflectance, the stage of vegetation development or the canopy structure heterogeneity among other, must be considered [5].
5. Most of the studies on biomass characterization in rangelands and grasslands by remote sensing are applied to tropical, arid and semi-arid environments. Grasslands in temperate climates are much less studied in comparison [5]. This study therefore contributes to the characterization of this type of environment.

### 4.2. Volumetric-based biomass

Although limited by the number of samples, our results show a good correspondence between the biomass measured in the field and the estimated volume with a very good precision (NRMSE < 10%). These results are comparable to or even better than similar studies, which have tested SfM approaches on UAV imagery to derive sward height and biomass in grasslands. These studies found a relatively good correspondence between canopy heights measured in the field and those modeled by SfM with R^2^ between 0.56 [41], 0.57 [42], 0.86 [44] and 0.90 [40], or in comparison with LiDAR data with an R^2^ of 0.62 [4]. However, the correspondence between the variables derived from these sward height models and the biomass measured in the field remains variable according to studies with R^2^ of 0.23 [4], 0.10 to 0.41 [43], 0.54 [42], 0.54 to 0.72 [38], 0.58 to 0.81 [41], 0.66 to 0.78 [40], and 0.93 [23]. The correlations obtained in this study therefore exceed those of existing studies with R^2^ between 0.93 (fresh biomass) and 0.94 (dry biomass).

Several limiting factors such as the low variability of biomass values in the datasets, the spatial resolution of UAV imagery, the density and variety of grasslands, or the presence of lodging may partly explain the variable correlation levels in certain studies [4, 40, 41, 43, 44]. However, the availability of a precise reference level (i.e. DTM) remains a key element to ensure the accuracy of the models, since measured vegetation heights are often <1m. This limitation is poorly addressed in the literature but could explain mixed results observed in certain studies. The present study, as well as existing ones, focuses on small areas (e.g. 5 x 5 m plots) in flat terrain for which precise geospatial data are available or can be measured relatively easily [40, 41, 43, 44]. However, at the field or farm scale, the topography can be highly variable, and precise data is more difficult to obtain. Ideally, a reference DTM would be generated beforehand when there is no vegetation, which is not possible in natural grasslands, for example.

The increasing availability of LiDAR data at regional scales is promising. However, the available point density is generally less than 3 points/m^2^ in Quebec [45] and does not allow generation of sufficiently-precise DTMs for this application. For example, in Quebec, the DTM generated by the government from a systematic LiDAR coverage of the territory has an altimetric error of up to 0.25 m (Ministère des Forêts, de la Faune et des Parcs, Pers. Comm.). In this study, tests were performed at the field scale (14 ha) using this dataset. The results did not allow detection of variations in vegetation height that were sufficiently precise to be applicable (results not shown). The integration of a LiDAR sensor on board UAVs is an interesting avenue for generating accurate DTMs. However, the availability of affordable sensors and the complexity of data processing may limit its applicability. The available studies also showed mixed results for grassland biomass modeling using a LiDAR equipped UAV [46].

### 4.3. GNDVI-based biomass model

Most of the previous studies looking at the relationship between VI and biomass used single regression on limited sets of data with little geographical and temporal variation. In the present study, data originate from a single field but across a whole four-month season of growth and include 14 UAV flights. Therefore, the variability of the data recorded is quite high and corresponds to various vegetation growth stages, ambient temperature, and meteorological conditions, which increases the external validity of the model. Moreover, the model was validated on an independent set of data with a very good concordance for fresh forage estimation (1.6% of cumulated error for regression and central tendency), whereas the concordance was lower for dry content estimation (10.5% error). The coefficients of determination of the linear regressions between predicted and measured values for the validation data set (R^2^ = 0.63 and 0.50 for fresh forage and dry content, respectively) were lower than for the training dataset (R^2^ = 0.80 and 0.66, respectively). Such reduction in the accuracy of the model with a validation dataset was also observed by Insua et al. [3], who found a good relationship between grass height in pasture and NDVI (R^2^ = 0.80) on experimental plots, but the relationship was poorer at a farm scale (R^2^ = 0.63). Lussem et al. [22], who have also validated a regression model between biomass and NDVI with an independent set of data, found similar coefficients of determination for the calibration and the validation datasets (R^2^ = 0.65 and 0.62, respectively). Therefore, the model developed in our study for the estimation of fresh forage biomass performed just as well as models from other studies on fresh forage biomass estimation. However, the estimation on a dry content basis was less accurate.

Considering the precision of the model, RMSE found in other studies using NDVI were between 0.057 and 0.063 kg DM/m^2^ for dry content [22, 24]. The RMSEs found in our study are higher, with 0.102 kg DM/m^2^ for dry content. The lower precision of our model could be explained by the high variability of our data, which is partly due to the extensive period of sampling. However, the amount of dry biomass in our study averaged 0.354 [0.060; 0.824] kg DM/m^2^ (mean [min; max]), which is higher than in the other studies where biomass averaged between 0.112 [0.002; 0.448] [24] and 0.28 [0.09; 0.49] kg DM/m^2^ [22]. Therefore, NRMSE of our model, which is 29%, is comparable to or even lower than other studies (20% [22] and 56% [24]) for the estimation of dry biomass, and even better for the estimation of fresh biomass (24%). Some studies using multiple regression based on several indicators such as VI, crop surface models and other indicators are able to achieve better performances with R^2^ > 0.90, and NRMSE ≤ 15% [3, 23]. However, these models are less practical because they are more complex and require multiple measurements or external source of data (e.g. soil, climate, farm management).

### 4.4. GNDVI-based vegetation cover classification

The second GNDVI-based approach uses thresholds of the VI values to establish vegetation cover classes. By combining information related to the studied system (e.g. pasture unit or bare soil unit), the season (e.g. date of clipping, start of growth), and stage of forage growth (i.e. duration in weeks), this approach allows the level of vegetation cover to be categorized in four classes, regardless of the period in the growing season. This approach is qualitative compared to the previous approaches but is probably more robust. Although our data did not allow us to validate this approach with field biomass measurements, the results presented in the control unit (see Fig 7) indicate a good agreement with the quantitative approaches. These thresholds were established using a statistical approach but could also be based on a supervised approach using field data in order to represent particular classes of height or density, for example.

### 4.5. Comparison and applicability of the methods

Overall, as shown in Fig 7, the three models tested in the present study allow characterization of biomass or vegetation cover providing very high-resolution data over the study area. These approaches can capture plot heterogeneity at fine spatial, and potentially temporal, resolutions. These approaches thus provide a major advantage over field approaches (e.g. biomass sampling, height stick, rising plate meter, rapid pasture meters) by providing information that is more complete spatially and temporally [38, 40, 44]. Although these approaches also require time and a level of specialized expertise [41], the level of information provided paves the way for various high-resolution spatio-temporal monitoring applications as well as full coverage at farm and ecosystem scales. The results of this study also show different levels of characterization between the approaches. Therefore, they would not be applied in the same context nor for the same purpose.

The volumetric-based biomass model is very precise but does not seem sensitive enough to differentiate vegetation from soil at low vegetation levels and does not provide information on vegetation health (e.g. piles of dead vegetation). It is also limited by the availability of an accurate DTM, and further studies are required to better understand the impact of variables such as spatial resolution or variety and density of grasslands. However, this model is not limited for fresh biomass greater than 0.5 kg/m^2^ (0.1 kg DM/m^2^), and its linear relationship with biomass is easier to model compared to the GNDVI-based model. The volumetric approach can be used to determine when a growing forage plot is ready for harvest but is limited in guiding rotation between grazing plots because of its low detection accuracy of low volumes. It can also be useful to characterize sites with high biomass and volume levels, such as highly-productive grasslands. When precise DTM are available, this approach is simpler to use and has a better generalization potential compared to spectral-based approaches.

The GNDVI-based biomass model performs with a good accuracy. However, the saturation of the GNDVI leads to a lower precision of the estimation as the amount of biomass increases, which limits its application for high biomass levels, especially for fresh biomass greater than 3 kg/m^2^ (0.6 kg DM/m^2^), which is equal to or greater than the usual yield observed for most forage productions [47]. On the other hand, GNDVI allows efficient discrimination between green short vegetation and soil. It can be applied to monitor vegetation growth and guide rotations between grazing plots. This approach can also be interesting for monitoring animal consumption, even though disturbance of the vegetation by certain species (e.g. sow trampling and rooting) can lead to high levels of uncertainty [27].

The GNDVI-based vegetation cover classification can be easily derived from the GNDVI-based biomass model and would target different applications. The classification approach allows better illustration and detection of large variations in vegetation cover. This classification approach potentially represents a simple, quick to use and generalizable tool for managing pastures. Such a model can be especially useful for the detection of pasture degradation by grazing animals in order to plan rotation between grazing plots. It could also be used to determine when a growing forage plot is ready for harvest or pasture, and the thresholds between classes could be modified to correspond to relevant amount of biomass used in production practices.

Several approaches have recently tested the combination of multiple information, by integrating non-spectral information (e.g. 3D, ultrasonic) with spectral information for the evaluation of biomass in grasslands [4, 43, 48]. These studies usually show higher performances of approaches based on spectral information alone compared to approaches based on 3D or ultrasonic information alone. The combination of the two provides mixed results ranging from an increase [4, 48] to a decrease [43] in the performance of the models. Although these approaches provide promising results, this combination of information often remains linked to the use of several sensors or the implementation of several data processing chains, which can complicate the production of information in an operational context.

## 5. Conclusions

The present study developed and compared different methods for evaluating forage biomass or cover using vegetation indices and volumetry. The methodology used allowed the estimation models to be trained and validated with a good level of confidence thanks to the high variability of the data recorded across a whole season, the selection of the best VI fitted to the data set used—in our case the GNDVI—and the validation of the GNDVI-based model on an independent set of data. Each method showed technical advantages and limitations that qualify them for different applications related to forage production, grassland monitoring or pasture management in a temperate climate. The GNDVI-based classification method looks promising for monitoring the vegetation cover degradation and simple and adjustable enough to be used by producers in a commercial context. The volumetric-based model showed a very good relationship with biomass but requires a reliable DTM. In that sense, the use of multi-sensor UAVs including LiDAR could be useful and would merit further consideration. Finally, further validation of the GNDVI-based model would be needed to confirm its efficiency and accuracy in measuring small biomass variations related to grazing by animals and determine if it could be used for the estimation of their forage intake.

## Supporting information

S1 Table Image datasets with their collection date, time, cloud conditions, and wind speed. (DOCX)

S2 Table Number of 3.5 × 3.5 m polygons used for vegetation cover classification based on the date of clipping and the forage growth duration. (DOCX)
