# Supplementary material for: Estimation of forage biomass and vegetation cover in grasslands using UAV imagery
Source: PLoS One. 2021 Jan 25;16(1):e0245784. doi: 10.1371/journal.pone.0245784 (PMC7833225; doi:10.1371/journal.pone.0245784)
Supplement: S1 Table — (DOCX) [file pone.0245784.s001.docx]

**S1 Table. Image datasets with their collection date, time, cloud conditions, and wind speed.**

| **Date** | **Median flight time (EDT)** | **Cloud conditions** | **Wind speed (km/h)^a^** |
| --- | --- | --- | --- |
| June 6th | 13:45 | Varying | 14 |
| June 13th | 10:00 | Varying | 20 |
| June 21st | 13:15 | Varying | 26 |
| June 27th | 09:30 | Sunny | 10 |
| July 4th | 15:30 | Sunny | 15 |
| July 11th | 10:45 | Varying | 10 |
| July 18th | 10:00 | Varying | 3 |
| July 25th | 10:15 | Cloudy | 6 |
| August 1st | 10:15 | Sunny | 10 |
| August 8th | 10:45 | Varying | 0 |
| August 15th | 09:45 | Sunny | 8 |
| August 29th | 12:00 | Varying | 3 |
| September 12th | 11:30 | Sunny | 13 |
| September 26th | 09:30 | Sunny | 0 |
