# Supplementary material for: Estimation of forage biomass and vegetation cover in grasslands using UAV imagery
Source: PLoS One. 2021 Jan 25;16(1):e0245784. doi: 10.1371/journal.pone.0245784 (PMC7833225; doi:10.1371/journal.pone.0245784)
Supplement: S2 Table — (DOCX) [file pone.0245784.s002.docx]

**S2 Table. Number of 3.5 × 3.5 m polygons used for vegetation cover classification based on the date of clipping and the forage growth duration.**

| **Clipping date** | **Forage growth duration (weeks)** | | | | | | | |
| --- | --- | --- | --- | --- | --- | --- | --- | --- |
|  | **Bare soil** | **1** | **2** | **3** | **4** | **5** | **6** | **Total** |
| May 23-25th |  |  | 5 | 7 | 1 | 1 | 1 | 15 |
| June 1st |  | 8 | 4 | 4 |  |  |  | 16 |
| June 6-8th |  | 6 | 5 | 5 | 5 | 5 | 6 | 32 |
| June 13-15th |  | 6 | 5 | 5 | 5 | 5 |  | 26 |
| June 20-22th | 2 | 1 | 1 |  | 5 | 4 |  | 13 |
| June 27th | 2 | 7 | 6 | 6 | 5 | 1 | 6 | 33 |
| July 4-6th | 6 |  |  |  | 5 | 3 | 6 | 20 |
| July 11-12th | 7 | 7 | 6 | 4 | 4 | 4 |  | 32 |
| July 18-20th | 7 | 1 | 1 | 1 | 1 |  | 6 | 17 |
| July 28th |  |  |  |  |  | 5 |  | 5 |
| August 1st | 6 | 1 | 1 |  | 1 |  |  | 9 |
| August 8th | 3 | 1 |  | 1 |  |  |  | 5 |
| August 15-17th | 6 |  | 1 |  | 6 |  | 6 | 19 |
| August 22th |  | 1 |  |  |  |  |  | 1 |
| August 29th | 5 |  |  |  |  |  |  | 5 |
| **Total** | **44** | **39** | **35** | **33** | **38** | **28** | **31** | **248** |
